# Endophthalmitis After Cataract Surgery: A Postoperative Complication

**DOI:** 10.7759/cureus.30110

**Published:** 2022-10-09

**Authors:** Akshad M Wadbudhe, Shivangi C Tidke, Pravin K Tidake

**Affiliations:** 1 Ophthalmology, Jawaharlal Nehru Medical College, Datta Meghe Institute of Medical Sciences, Wardha, IND

**Keywords:** prevention, visual acuity, vitreous, vitritis, inflammation

## Abstract

Endophthalmitis is a condition of the eye caused due to complications in cataract surgery. The extent of this complication can be from minor to very serious, leading to a permanent loss of light perception. It is generally an inflammation of the fluids present in the anterior and posterior chamber of the eye, consisting of vitreous and aqueous fluid. The inflammation is due to the infection of these fluids after their exposure during or after the cataract surgery. In today's situation, patient surgery is the most frequently preferred for the correction or treatment of the cataract. There are various factors causing endophthalmitis in cataract surgery. This condition occurs mostly by the entry of infective bacteria such as staphylococcus, gram-negative organisms, and streptococcus species. As well as fungi like aspergillus and candida. Cataract surgery has many risk factors that can be divided into preoperative, intraoperative, and postoperative phases. The most common symptom of this condition is pain in the eyes and redness, which sometimes leads to purulent discharge, causing decreased vision or loss of eyesight. The increasing inflammation of the vitreous fluid is the main identification of the condition. There is a surge of inflammatory cells in the space of the vitreous fluid. The condition can be classified into two types which are exogenous and endogenous. In these types, subtypes explain the postoperative complications of the disease. It is a rare condition, and the percentage of it occurring as a postoperative complication is very low. It generally targets the old age group of people. This narrative review article explains endophthalmitis as a postoperative complication of cataract surgery and its treatment modalities. The terms endophthalmitis, postoperative, cataract surgery, complications, and vitreous humor were used for the review article in PubMed.

## Introduction and background

Endophthalmitis is the inflammation caused due to the infection of the fungi and bacteria in the eye, including the fluids aqueous and vitreous humor [1]. It is a rare type of complication which occurs in a few patients after cataract surgery [2]. In this condition, the organisms enter the eye through some accident or trauma, by different surgeries of the eye, or some infectious diseases of cornea or structure near the vitreous humor. Endophthalmitis can be caused by some internal factor or reaction, but the majority of cases are by the outside agent. The inflammation does not help in the increasing of pathogens in blood. These types of cases are critically important to treat and take care of. If the treatment is late, it could lead to complete blindness. in a maximum number of cases the patients will have common complaints of pain, redness of the eye, photosensitive, and progressive decreasing of vision. The inflammation affects both aqueous and vitreous humor present in the eye. The white blood cells are present in the humor of the eye suggesting the inflammation be infectious. The most important treatment modalities for such cases are antimicrobial therapy. Vitrectomy is also the leading way of the treatment of endophthalmitis but leads to the toxicity of drugs in the ocular region. All over the world, the most commonly done ocular surgeries done is cataract surgery which is mostly followed by the complication of acute post-cataract endophthalmitis. Fungi are the major cause of endophthalmitis in most tropical countries including India. But in all the other countries such as Europe and USA, maximum prevalence is contributed by the bacteria such as streptococci, Staphylococcus aureus, and various other gram-positive cocci and gram-negative bacilli. Various non-infectious causes for endophthalmitis include foreign bodies which are retained in the intraocular region, exogenous toxins, and various allergic reactions [3].

To confirm the diagnosis of endophthalmitis various diagnostic procedures are used like culture methods. To detect the causative pathogen involved, molecular diagnostic techniques have been implicated which significantly improved the sensitivity. The treatment modalities include vitrectomy along with the use of intravitreal antibiotics. In allergic cases, steroids may be intravitreally injected. Specifically, the antimicrobials which are broad spectrum do not work in this complication. The visual acuity of a patient after the surgery differs on the pathogens affecting the eye. If the organism is bacteria like streptococci the recovery is very low, on the other side the fungal infection would have more chance of recovery of vision. The fungal infection affects the choroid of the eye and then starts spreading across the humor. The chance of infection by fungi is very low. Endophthalmitis can also be caused by secondary causes like mold infection in the cornea known as keratomycosis. As the mold starts to multiply and move towards aqueous humor [3]. This complication risk increases with age and is associated more with males and less with females. Some other progressive risk factors are type one and type two diabetes, high blood pressure, some carcinoma, and stroke [4].

## Review

Methodology

The terms endophthalmitis, postoperative, cataract surgery, complications, and vitreous humor were used for the review article in PubMed. The review articles were filtered between the year 2007-till date. The appropriate articles were also taken from google scholar by the terms mentioned above.

Classification of endophthalmitis

**Figure 1:**
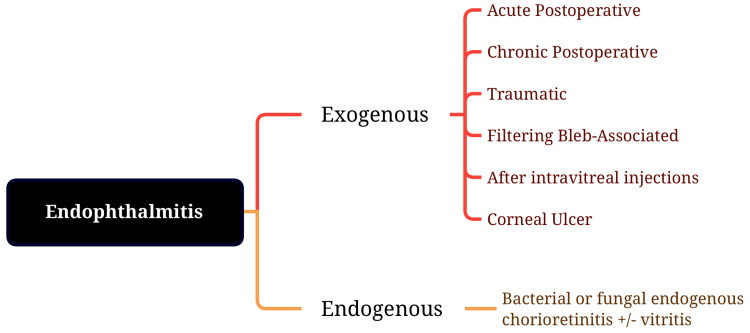
Classification of endophthalmitis

The classification of endophthalmitis is depicted in Figure 1. It is divided into two major groups: Exogenous and endogenous. Exogenous endophthalmitis can be divided into acute postoperative, chronic postoperative, corneal ulcer, after intravitreal injections, traumatic and filtering bleb-associated [5]. Endogenous endophthalmitis can be divided into fungal endogenous chorioretinitis with or without vitritis and bacterial endogenous chorioretinitis with or without vitritis [6].

In these many types, postoperative endophthalmitis is divided into two types: acute and chronic. The differences between acute onset and chronic onset of endophthalmitis are given briefly in Table 1 [7].

**Table 1:** Difference between acute endophthalmitis and chronic endophthalmitis

Features	Acute onset Endophthalmitis	Delayed/ Chronic onset Endophthalmitis
Time	within 6 weeks after surgery	after 6 weeks of surgery
Causative Agent	Bacteria: Group B Streptococci	Bacteria: Propionibacterium acnes Fungi: Aspergillus and Candida
Symptoms	Pain , diminished vision, Redness of conjunctiva, watering, photophobia	Mild pain gradual progressive diminished vision
Prevalence	More common	Rare

Acute postoperative endophthalmitis

Acute postoperative endophthalmitis is the sudden onset of inflammation caused by the infection inside the eye caused by microorganisms during surgery or post-surgery [8]. It mostly results in a visual loss if not diagnosed and treated at the right time [9]. The complications which are seen in less than six weeks are considered at the acute onset of the postoperative endophthalmitis. When the incision is given on the cornea for surgery, it cannot be sutured, which increases the risk of endophthalmitis. Other risk factors which increase the complication of acute postoperative endophthalmitis are considered to be high blood pressure, hyperglycemia, old age-related issues, immunocompromised patients, chronic blepharitis, people with contact lenses, and intraocular lens surgeries [10].

It is identified by the inflammation of the fluid present in the eye. In most cases of such complications, 70% are of acute onset postoperative endophthalmitis. Some common organisms causing it are gram-negative staphylococcus [11]. The staphylococcus epidermidis is the main organism responsible for the infection in the conjunctiva growth of bacteria. In this condition, subluxation of the lens or breakdown of the lens is a major factor. In hyperglycaemic conditions of a patient with old age, there is a slight difference in increased frequency of the complication. A number of people in some hospitals found that the heat in the surroundings and the moisture in the air has a significant effect on complication after the surgery [12].

Symptoms

The symptoms that the patient presents with are decreased vision, redness of conjunctiva, pain, edema, sensitivity to light, and excessive secretion of tears [13].

Signs

There might be an accumulation of neutrophils and fibrins that settle ventrally within the anterior chamber, with no clear view, only light can be recognized. There can be confusion between the inflammation complication of post-surgery and other blockages of the vision, like deformities, and more preference should be given to the complication caused by the inflammation post-surgery. This is because if the diagnosis is not right and treatment gets late, it will lead to massive complications and the patient condition worsening [14].

Epidemiology

A lot of studies are done on the epidemiological factors of the cases of acute onset of endophthalmitis. One of the reports states that from 2009 to 2018, there were around 7,776 cases of the acute onset of endophthalmitis. The patient’s age was in the range of 63-83 years which comes in the group of old age. Based on gender, there were an equal number of patients on both sides. The rate of complications due to Cataract surgery decreased rapidly in consideration of the other cornea surgeries and vitreous interventions [15].

Evaluation

The evaluation of the size of the surgical eye compared to other eye after the surgery as there might be differences in them which give idea about the defect/problem in the eye; secretion of any substance like pus discharge mucoid fluid from the incised area indicates the growth of the infectious organisms; the suture has a gap between them becoming the main entrance for the substance to enter leading to complication [16].

Visual Acuity

The visual acuity of the patient with acute onset of endophthalmitis is very low, and they cannot get a clear vision at 3/60 of Snellen's chart. Some patients have a light perception factor where they can identify the light rays and from which direction it is coming.

Differential Diagnosis

Some of the differential diagnoses are left-over broken parts of the lens, old infection of the uvea, which increased due to surgery, rapid loss of the blood in the vitreous humor, and toxic anterior segment syndrome. These are differentiated as the effect of the inflammation is seen after a day of the surgical procedures. In contrast, the toxin effects are rapid and cause massive swelling in the orbital region of the eye [17].

Treatment

The treatment starts with the antimicrobial drug given inside the vitreous humor, vancomycin 1mg in 0.1mL simultaneously with other drugs, ceftazidime 2.25mg into 0.1mL. These drugs should be given simultaneously but with different needles and vials. If the ceftazidime is not available substitute for it is amikacin 400μg into 0.1mL. Some of the drugs which can be applied to the outer part of the eye are ciprofloxacin for 60 minutes, or the alternative for it is the combination of tobramycin and cefazoline for 60 minutes with muscarinic receptor blockers like homatropine every six hours. These drugs are changed with the response they cause. Pars plana vitrectomy can be done. Steroids are also administrated in the management after 24-48 hours. The route for these steroids is oral and is given with oral antimicrobials [18].

Prevention

The prevention of this complication can be given in the form of the preparation of a 5% of the povidone-iodine solution. Also, the prevention of antimicrobials is given. The patient's condition should be stable and free from any other infection in other parts of the body. Samples for testing before the cataract surgery should be taken carefully. The preoperative risk factors should also be considered before the surgery, and There are not many studies about prevention [19].

Chronic postoperative endophthalmitis

Chronic postoperative endophthalmitis is the long-term inflammation caused by the infection of the organism, which occurs after six weeks of the cataract surgery. This is because the diagnosis is done late or may be the effect of using corticosteroids for a long time. It is generally underestimated as inflammation with no infection of the uvea [20]. The microorganisms which are responsible for acute endophthalmitis are different from the organisms which cause chronic endophthalmitis [21]. The organism mostly responsible for chronic endophthalmitis is the Propionibacterium family of bacteria. It is not a frequent complication but causes severe damage to the eye leading to blindness [22]. The chronic onset of the endophthalmitis is the intraocular inflammation in which the parts of the eye that get inflamed are the vitreous chamber and aqueous chamber. Some of the other parts that can also get affected by this inflammation are the choroid, retina, cornea, conjunctiva, etc [23]. the chronic onset of endophthalmitis caused by the fungal infection is generally caused by the organism known as candida species. Most infections in the human body are caused by the candida species [24].

Symptoms

Pain and agitation, low vision to complete loss of vision, orbital edema, and redness of the conjunctiva [25].

Signs

The white blood cells are found in the vitreous chamber as the protective mechanism of the immune system. Accumulation of neutrophils and fibrins that settle ventrally within the anterior chamber. Subluxation of the lens and the left-over particle of the lens is present in the vitreous humor. Plaque is seen in the eye of the organism [26].

Evaluation

The evaluation of chronic endophthalmitis is mostly with clinical thought with the other test findings. Biopsy of the vitreous chamber by the needle aspiration method. Tests like gram stain, immunofluorescence, and other tests for microorganism findings these types of culture require a week or more to grow as the organisms are very slow growing in nature [27]. The plaque formed in the eye is also used as a special type of culture for organisms to grow. The inflammation can also occur due to the implant placed in the eye, which starts to get irritated and cause it, so checking any implants is also a very important part of evaluation [28].

Epidemiology

Many studies are done on the epidemiological factors of the cases of chronic onset of endophthalmitis. One of the reports states that cases vary from 0.01% to 0.367% of cataract surgery. Some studies reported that the ratio of the acute onset of the endophthalmitis to the chronic endophthalmitis is in the range of 5:1 to 2:1. The rate of complication is higher in the cataract surgery procedure whereas less in the other surgeries of the eye, i.e., 0.367% of the total surgeries. The complication rate of cataract surgery is five patients per thousand patients. With the increase in the precautions and prevention of the condition, the chronic endophthalmitis rate has decreased to 0.017% [29].

Visual Acuity

The visual acuity of the patient with the chronic onset of endophthalmitis is better than that of the acute onset of endophthalmitis, with over half of the patients having 20/40 in Snellen's chart; another half of them have a visual acuity vision less than this to worse than 5/200 [30].

Differential Diagnosis

The chronic onset of endophthalmitis is mostly misunderstood with the long-term inflammation of the infection, less type of the uvea, rapid loss of blood in the vitreous humor, and toxic anterior segment syndrome. The differentiating features of postoperative endophthalmitis and toxic anterior segment syndrome are mentioned in Table 2.

**Table 2:** Difference between postoperative endophthalmitis and toxic anterior segment syndrome

Features	Postoperative Endophthalmitis	Toxic Anterior Segment Syndrome
starts in	within a week or more	in a day
caused by	infection caused by microorganisms like fungal, bacterial, and some type of pathogen	entry of toxic agent leading to non-infectious reaction.
symptoms	blurred vision and pain	decreased visual acuity and corneal oedema
signs	hyperemia	higher intraocular pressure
differentiating factors	involves vitreous chamber	limits to anterior chamber
treatment	Intravitreal and t opical Antibiotics	Corticosteroids and c ulture anterior chamber

These are differentiated as the effect of the inflammation is seen after a day of the surgical procedures. In contrast, the toxin effects are rapid and cause massive swelling in the orbital region of the eye. The condition of retinoblastoma in children also mimics the chronic onset of endophthalmitis [31].

Treatment

The chronic onset of endophthalmitis is not found as frequently as the acute onset of endophthalmitis and is caused by the microorganism of the family Propionibacterium, which comes as bacteria; hence the treatment is not to go for direct surgery but to start the patient with antimicrobials and other supportive drugs with the route of administration as directly into the vitreous chamber [32]. The name of the drug is vancomycin. Pars plana vitrectomy can be done. Some drugs are given through oral routes, which are moxifloxacin [33]. Vitrectomy is one of the methods used to remove the infection-causing organisms and the toxins released by them. This could lead to the displacement of the retina for limited time and give a better space for distribution of the drugs induced in eye. In the scarcity of vitreous humor, the drugs can cause a toxic reaction as a side effect. Vitrectomy is done with the administration of intravitreal drugs for better management. There is not much management required for the chronic onset of endophthalmitis, and antimicrobial therapy works on the condition [34].

Prevention

The prevention of the chronic onset of endophthalmitis is to reduce the risk factors of the operation and infection. The condition of the patient should be stable and free from any other infection in any other part of the body [35]. Samples for testing before the cataract surgery should be taken carefully. Povidone-iodine preparation is used with other therapy of antimicrobials like ciprofloxacin and norfloxacin [36].

## Conclusions

In conclusion, endophthalmitis, a postoperative complication of cataract surgery, should always be considered an important factor for the complications and further loss of vision. Endophthalmitis is divided into two types on the basis of the time of causing an inflammation: the acute onset of endophthalmitis, which is considered inflammation within six weeks of cataract surgery, and the chronic onset of endophthalmitis, which is considered inflammation after six weeks of the cataract surgery. The acute onset of endophthalmitis occurs more often as the organisms tend to infect as soon as possible and have a greater impact on the eye. This leads to higher chances of causing blindness if it gets diagnosed late or is misdiagnosed. In other types, the organisms are slow and react in the eye slowly, which takes more time to show the symptoms. Patients with an immunocompromised body, diabetes patients, hypertensive patients, and patients with previous surgical intervention in the eyes must be more careful and cautious about the complications like these. The best way to manage the condition is by giving antimicrobial therapy and taking precautions like preoperative risk factors. The doctors performing such types of surgeries must investigate the complications of such cataract surgeries and consider the differential diagnosis of the inflammation of an eye.
